# Supplementary material for: Radiologic Evaluation of Uterine Vasculature of Uterus Transplant Living Donor Candidates: DUETS Classification
Source: J Clin Med. 2022 Aug 8;11(15):4626. doi: 10.3390/jcm11154626 (PMC9369657; doi:10.3390/jcm11154626)
Supplement: Supplementary file 1 [file jcm-11-04626-s001.zip › Supplementary Figures S1-S18.pdf]

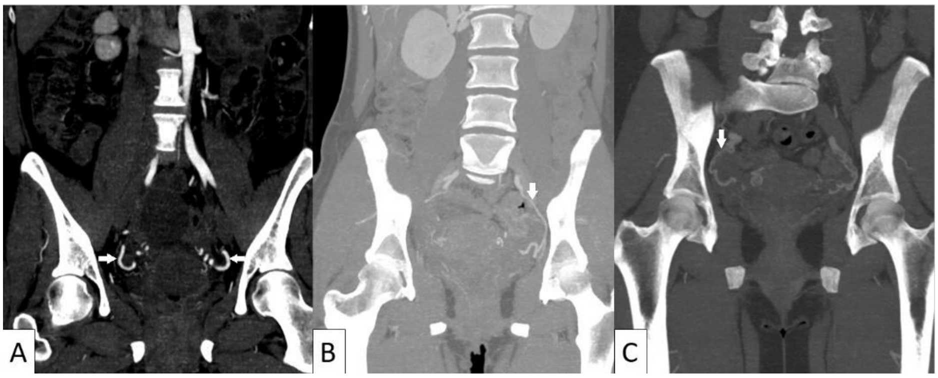

Figure S1: Uterine arteries. Bilateral UAs grade A (A, arrows); left UA grade A-minus (B, arrow); right UA grade A-minus (C, arrow).

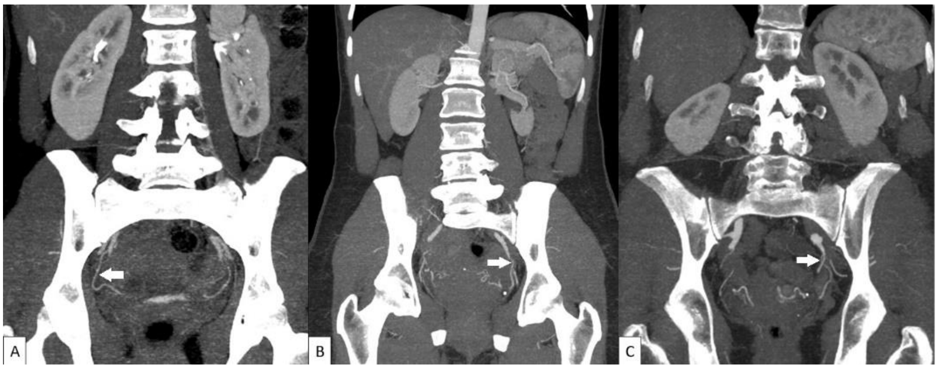

Figure S2: Uterine arteries. Right UA grade B (A, arrow); left UA grade B-minus (B, arrow); left UA grade B-minus (C, arrow).

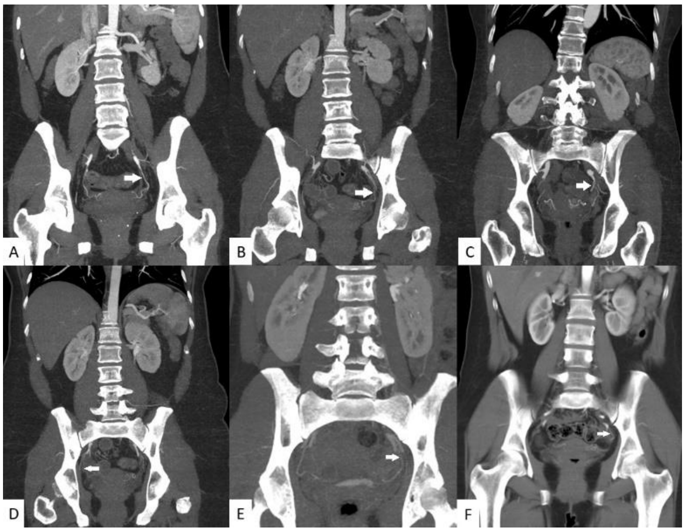

Figure S3. Uterine arteries. Left UA graded C (A, arrow); left UA graded C-minus (B, arrow); left UA graded C-minus (C, arrow); right UA graded C-minus (D, arrow); left UA graded C-minus (E, arrow); left UA graded C-minus (F, arrow).

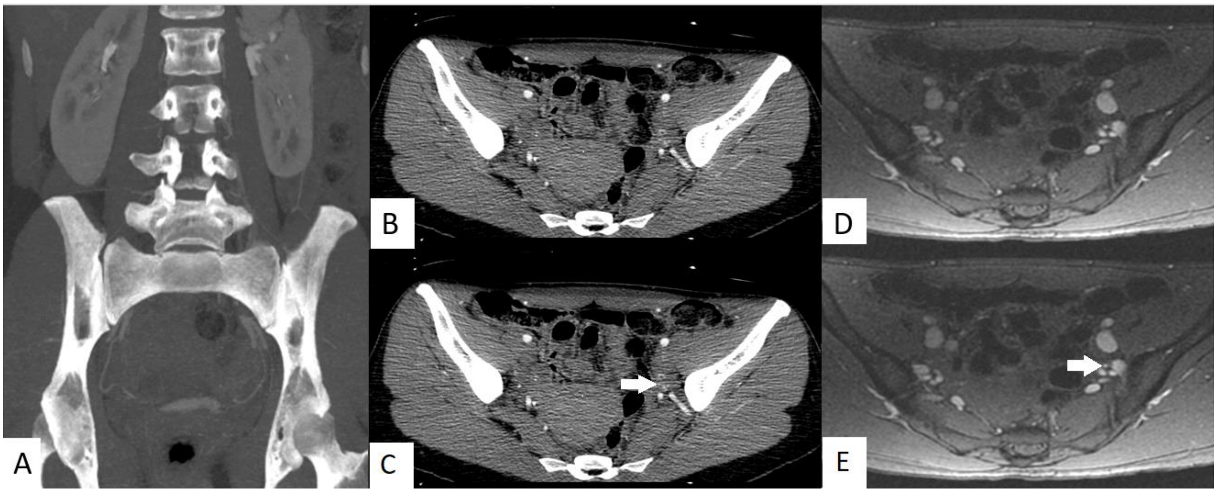

Figure S4: Uterine arteries. Left UA is called C-minus (A) and is not seen at its origin on CTA (B, C, arrow) due to its hyperdynamic pulsation but it is seen as completely normal on MRA (D, E, arrow).

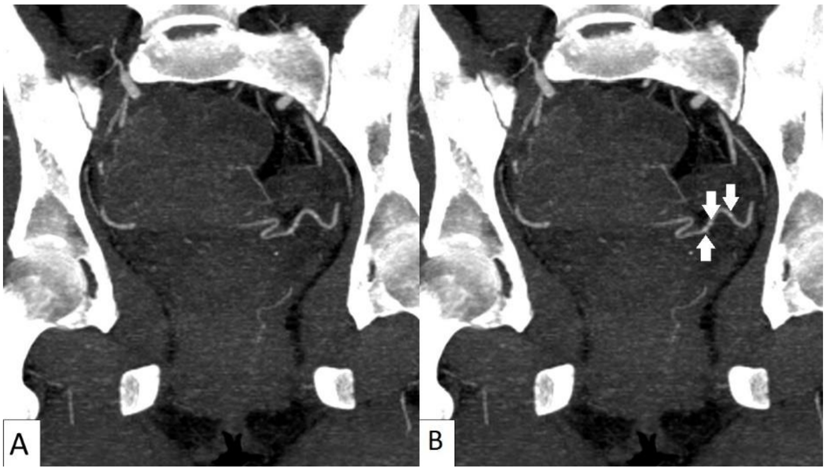

Figure S5: A left uterine artery demonstrating a moderate degree of atherosclerosis (A), highlighted by arrows in (B).

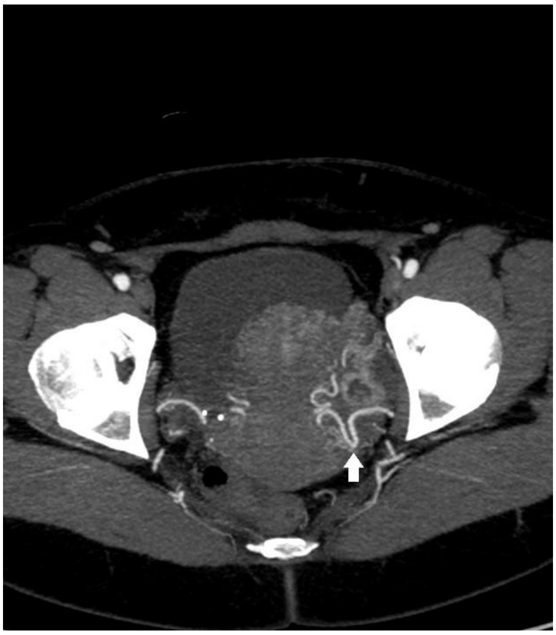

Figure S6: A left uterine artery demonstrating a "hairpin" turn (arrow), which is a risk factor for thrombosis.

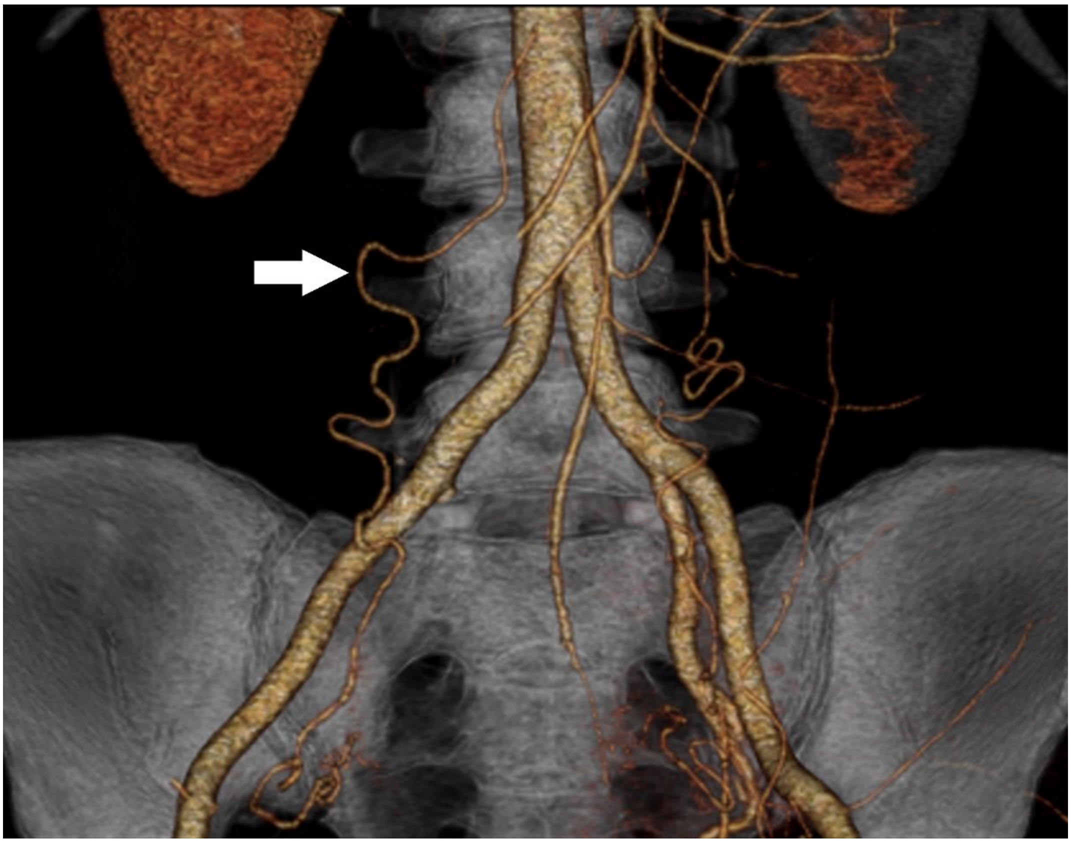

Figure S7: Arterial variation. No right uterine artery is present, only the right ovarian artery (arrow) is seen in reconstructed CTA.

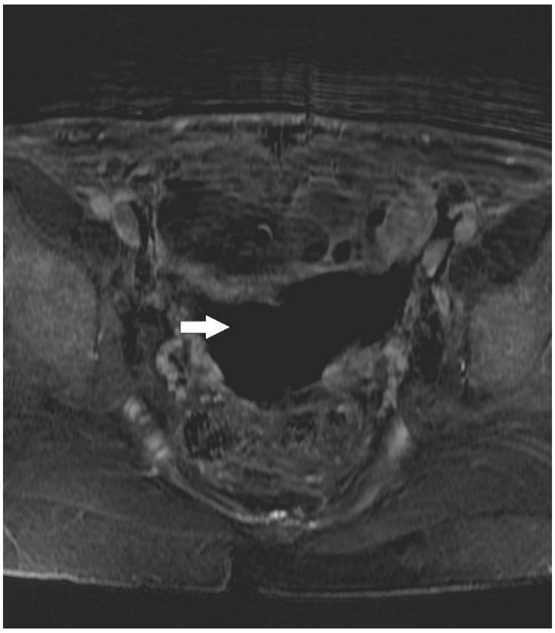

Figure S8: A non-viable uterus (arrow) without enhancement in MRA.

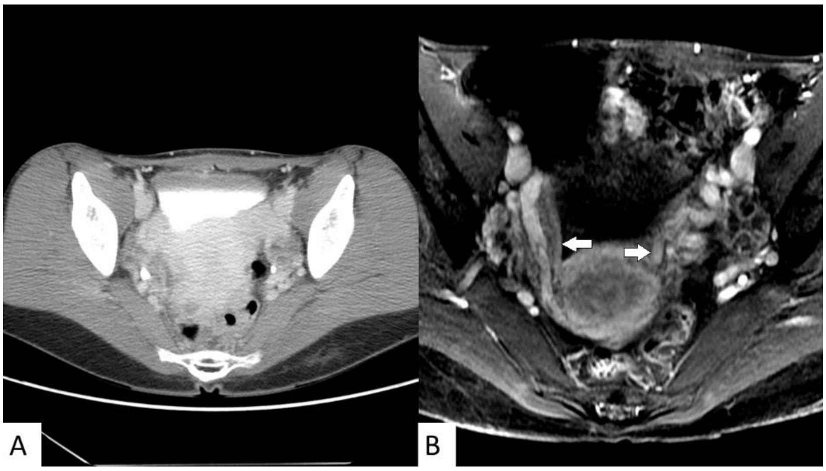

Figure S9: Superior uterine veins bilaterally graded A were not visible on CTA (A) but were perfectly well visible on MRA (B, arrows).

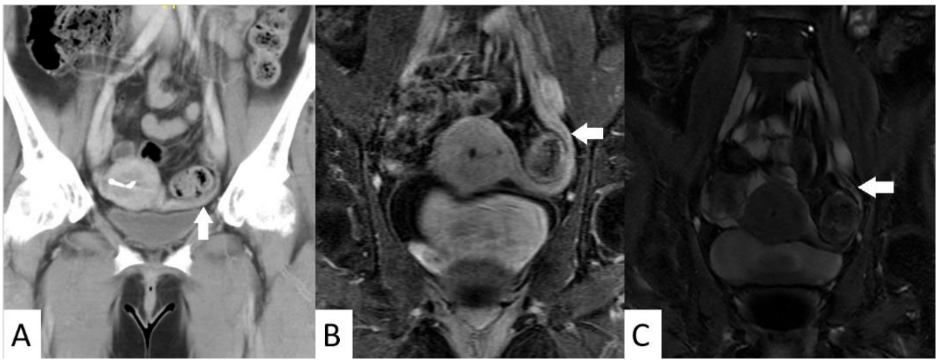

Figure S10: A left ovarian vein with fenestration visible next to the colon (A–C, arrows).

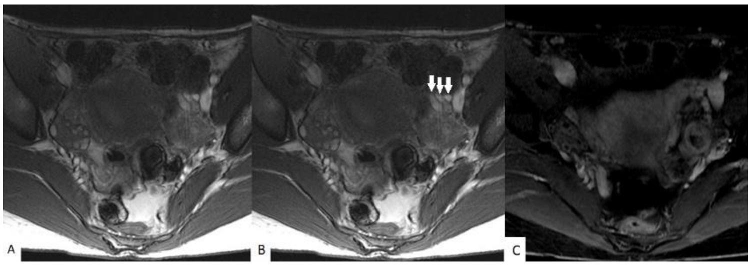

Figure S11: Three A-graded superior uterine veins visible with cardiac-gated images (A), highlighted with arrows (B), while post-contrast images suggest only two veins (C).

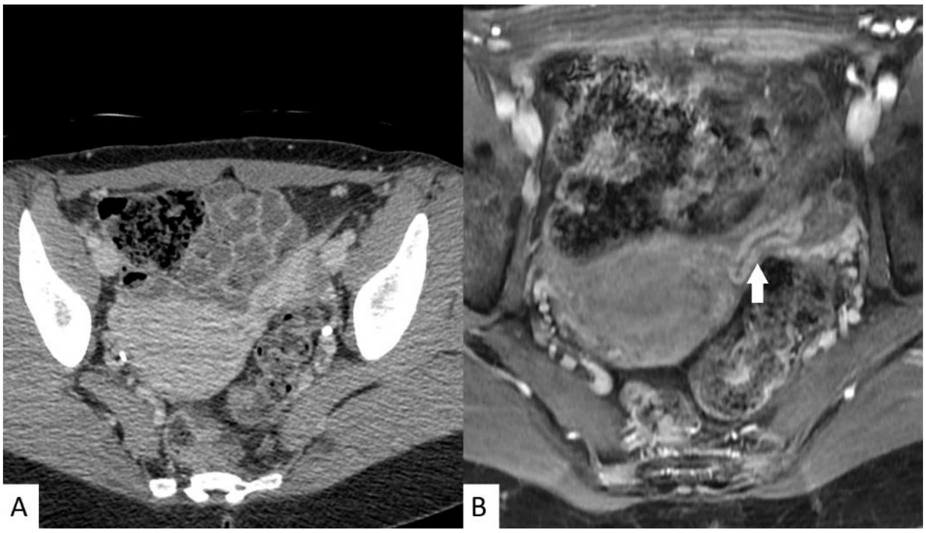

Figure S12: A fenestrated left superior uterine vein barely visible with CTA (A), but perfectly visible on MRA (B, arrow).

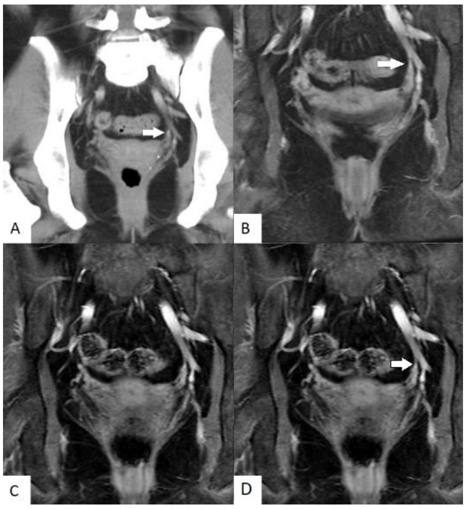

Figure S13: A left inferior uterine vein graded B-minus on CTA (A, arrow), and MRI (B–D, arrows).

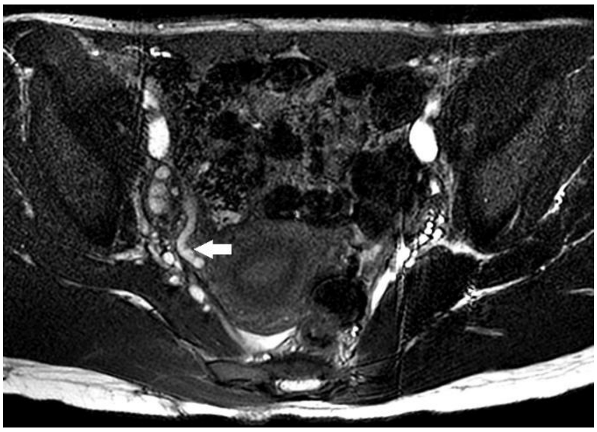

Figure S14: A right inferior uterine vein (arrow) graded B visualized with MRA.

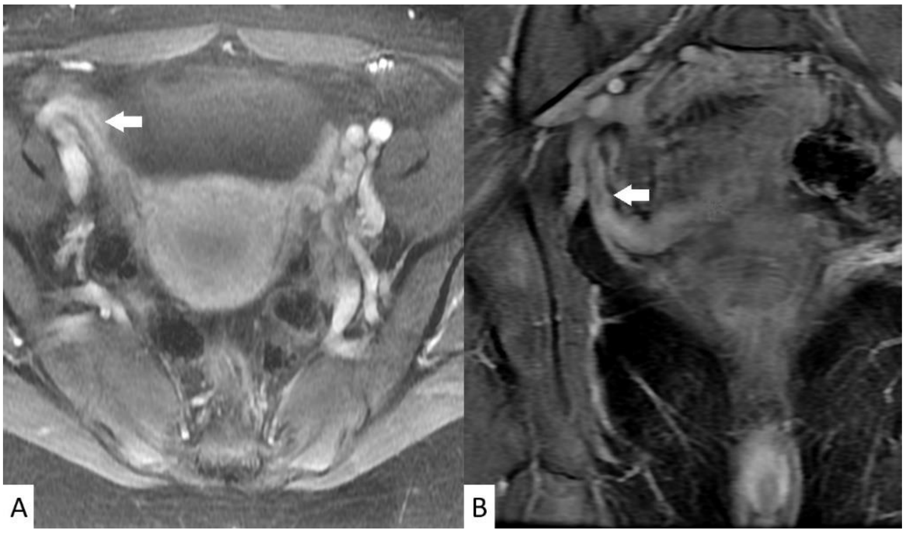

Figure S15: Fenestrated veins. Right superior uterine vein with fenestration graded A (A, arrow), right inferior uterine vein graded A with possible fenestration (B, arrow).

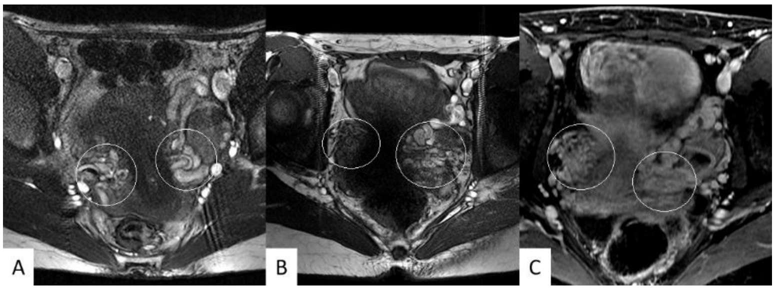

Figure S16: Plethora. Multiple varicose veins around the uterine cervix (A–C, circles).

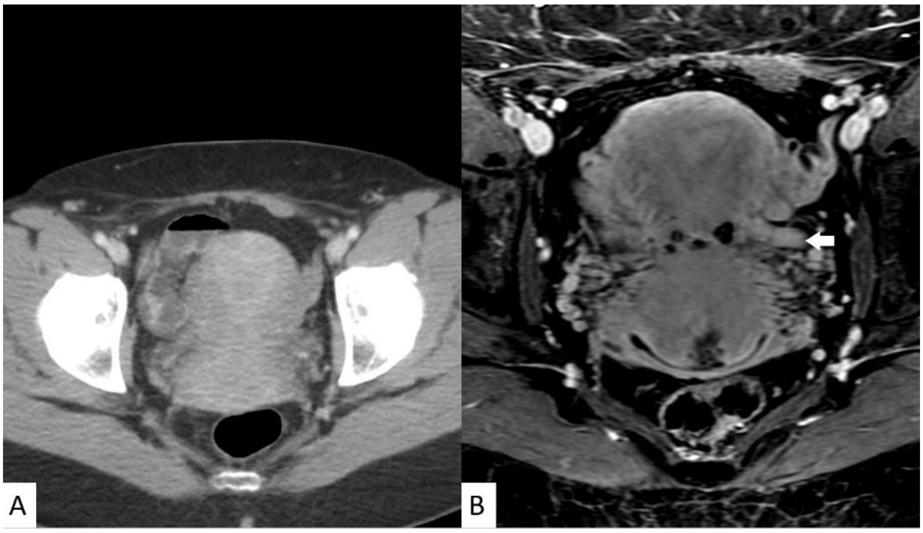

Figure S17: Inferior left uterine vein not visible on CTA (A), perfectly visible on MRA (B, arrow).

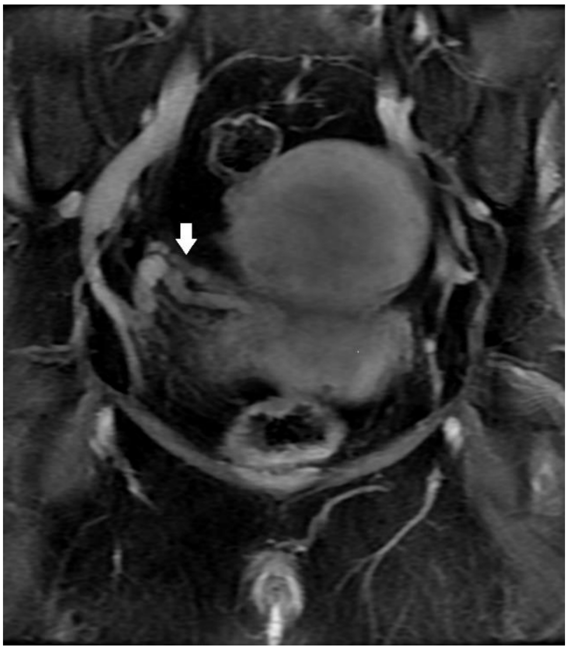

Figure S18: Right inferior uterine vein grade A. The vein was not procured because it was located too deep in the pelvis (A, arrow)
