# Supplementary material for: Radiologic Evaluation of Uterine Vasculature of Uterus Transplant Living Donor Candidates: DUETS Classification
Source: J Clin Med. 2022 Aug 8;11(15):4626. doi: 10.3390/jcm11154626 (PMC9369657; doi:10.3390/jcm11154626)
Supplement: Supplementary file 1 [file jcm-11-04626-s001.zip › Supplementary Table S1.pdf]

Table S1. Protocol of CT angiography and MRA evaluation of uterine living donor candidates as used in DUETS.

Imaging	Parameters used	Protocol, recommendation
CT angiography	• Scanner: 32-slice GE LightSpeed Pro (GE Medical Systems, Chicago, IL, USA)<br>• Intravenous contrast: weight-based low-osmolarity nonionic intravenous iodinated contrast (Omnipaque; GE Healthcare, Chicago, IL, USA)<br>• Scanner: 32-slice GE LightSpeed Pro (GE Medical Systems, Chicago, IL, USA)<br>• Intravenous contrast: weight-based low-osmolarity non-ionic intravenous iodinated	• Source images in the axial plane using automatic modulation at 0.625 mm thickness<br>• Scan in the arterial phase using bolus triggering<br>• Repeat axial images in the venous phase<br>• Axial reformats at 2.5 mm slice thickness<br>• Sagittal and coronal reformats at 5 mm slab thickness<br>• Axial, sagittal, and coronal maximum-intensity projection images at 10 mm slab thickness
MR pelvis and angiogram	• Scanner: 1.5T Discovery MR 750W or Signa HD XT (GE Medical Systems, Chicago, IL, USA)<br>• Contrast: weight-based double-dose intravenous Gadobutrol (Gadavist; Bayer Healthcare Pharmaceuticals, Montville, NJ, USA)	• Axial FIESTA (optional axial FIESTA fat saturation) 4 mm slice<br>• thickness with 4 mm gaps through the pelvis<br>• Axial two-dimensional time of flight FSPGR angiogram at 1.4- and 0.9-mm slice gaps<br>• Axial pre-contrast VIBE images at 2 mm thickness and 1 mm gaps<br>• Axial postcontrast images in the arterial and venous phases<br>• Delayed images at 4–6 min post-injection and reconstructed maximum-intensity projection rotation and tumble images<br>• All postcontrast images obtained with 4 mm slice thickness and 2 mm gaps<br>• Additional sagittal and coronal postcontrast VIBE images recommended especially in the venous phase

		• Optional: axial HASTE (5 mm thickness and 5 mm gap)
Abbreviation: CT, computed tomography; MR magnetic resonance; FIESTA: fast imaging employing steady-state acquisition; FSPGR, fast spoiled gradient; HASTE, half-Fourier acquisition single-shot turbo spin-echo; PRF, pulse repetition frequency; VIBE, volumetric interpolated breath-hold sequence.		
